# Demonstration of Quantitative Mid-Infrared Hydrocarbon Aerosol Absorption Spectroscopy Using a Dual-Cell Photoacoustic System

**DOI:** 10.1021/acs.analchem.6c03317

**Published:** 2026-08-27

**Authors:** Jamshed Saeed Shah, Abdul Rahman, Rana Khawar Ashfaq, Muhammad Haseeb Raza, Bálint Leits, Imola Urbán, Bálint Fodor, László Makai, Peter Petrik, Muhammad Qasim Mehmood, Gábor Szabó, Zoltán Bozóki, Cheng Tung Chong, Tibor Ajtai

**Affiliations:** † Department of Optics and Quantum Electronics, University of Szeged, 9. Dóm square, Szeged, H-6720, Hungary; ‡ HUN-REN-SZTE Research Group for Photoacoustic Monitoring of Environmental Processes, Dóm ter 9, Szeged, 6720, Hungary; § 249916 Semilab Semiconductor Physics Laboratory Co. Ltd., Prielle Kornélia u. 2, Budapest H-1117, Hungary; ∥ Institute of Technical Physics and Materials Science, HUN-REN Centre for Energy Research, Budapest, 1121, Hungary; ⊥ Department of Electrical Engineering, University of Debrecen, Bem Square 18, 4026 Debrecen, Hungary; # Department of Electrical Engineering, 434888 Information Technology University of Punjab (ITU), Lahore 54000, Pakistan; ○ China-UK Low Carbon College, 12474 Shanghai Jiao Tong University, Yinlian Rd, No. 3, Pudong, 201308, China

## Abstract

We report the development and demonstration of a simplified and robust photoacoustic (PA) technique by integrating an External Cavity Interband Cascade Laser (EC-ICL) with a dual PA cell configuration for the quantitative characterization of hydrocarbon aerosols in the mid-infrared range of 3.30 to 3.55 μm. The EC-ICL source was selected for its stable optical output, compact design, and low power consumption. By flowing a known gas through one PA cell, a wavelength-dependent transfer function (TF) is established and subsequently used to retrieve absolute absorption coefficients from the analyte aerosol measured in the second PA cell. As a proof of concept, propane was employed as the reference gas and diethyl-hexyl-sebacate (DEHS) aerosols as the analyte. Validation against bulk ellipsometry demonstrates a mean deviation of approximately 2.1% between the photoacoustically derived absorption coefficients and Rayleigh-theory predictions, establishing the quantitative accuracy of the transfer-function methodology. The proposed approach eliminates the need for external power normalization and frequent calibration procedures, providing a practical framework for quantitative mid-infrared aerosol absorption spectroscopy with applications in atmospheric aerosol characterization, source apportionment, and industrial emission monitoring.

## Graphical abstract

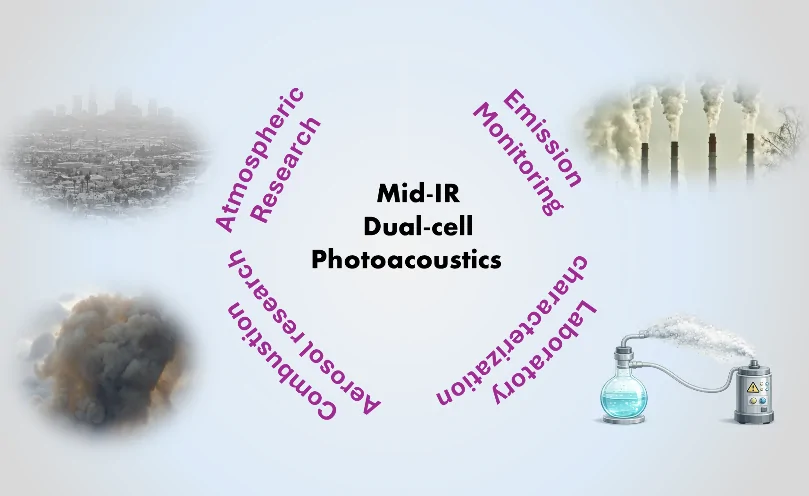

## Introduction

The mid-infrared spectral region (2.5–25 μm) hosts fundamental vibrational resonances of virtually all organic molecules, making it the fingerprint domain par excellence for chemical sensing.^1^ Within this region, the 3.3–3.55 μm window corresponds to the C–H stretching manifold—the signature transition of aliphatic hydrocarbons that dominate petroleum-derived fuels, lubricants, and their atmospheric degradation products.^2^ Quantitative measurement of aerosol absorption in this spectral band is therefore essential for applications ranging from real-time leak detection in oil and gas infrastructure to source apportionment of combustion aerosols and assessment of their direct radiative forcing.^3,4^ Yet despite its importance, a simplified methodology for obtaining quantitative absorption spectra of aerosols in this critical region has remained elusive. Quantitative aerosol absorption measurements remain challenging despite their importance for atmospheric chemistry, climate studies, and industrial monitoring. Most established aerosol absorption techniques operate in the ultraviolet, visible, or near-infrared spectral regions and primarily provide bulk optical properties rather than chemically specific molecular information. Moreover, the quantitative characterization of hydrocarbon-containing aerosols in the fundamental C–H stretching region remains comparatively underexplored despite the rich chemical information available in this spectral window. The 3.30–3.55 μm region is particularly attractive because it contains fundamental C–H stretching vibrations, providing significantly stronger and more chemically specific absorption signatures than the overtone and combination bands typically observed in the near-infrared.

Photoacoustic spectroscopy (PAS) offers unique advantages for aerosol in situ absorption measurements: high sensitivity, wide dynamic range, zero background signal, and—critically—insensitivity to light scattering, which plagues conventional transmission-based absorption techniques.^5–7^ The photoacoustic (PA) signal originates from periodic heating of absorbing species following periodic illumination, generating acoustic waves detectable with sensitive microphones. Because the PA signal scales linearly with the absorbed optical power, PAS is particularly attractive for detecting weakly absorbing species, where conventional path-length-dependent absorption techniques become limited.^8^ Recent years have witnessed significant advances in compact and high-sensitivity photoacoustic sensing platforms. Resonance-enhanced photoacoustic architectures have demonstrated remarkable detection sensitivity while maintaining compact instrument footprints, making them attractive for portable and field-deployable applications.^9^ In parallel, fiber-optic photoacoustic sensors have emerged as promising alternatives to conventional microphone-based systems, offering immunity to electromagnetic interference, remote sensing capability, and flexible system integration.^10,11^ These developments highlight the growing trend toward miniaturized, robust, and highly sensitive photoacoustic instrumentation for environmental monitoring, industrial process control, and chemical sensing.

PAS has been extensively applied in aerosol research for measuring absorption coefficients of atmospheric aerosols such as black carbon, brown carbon, and mineral dust, providing critical data for climate models and radiative forcing assessments. PAS has also been employed in source apportionment studies, where the Absorption Ångström Exponent (AAE) derived from photoacoustic measurements serves as a marker for distinguishing emission sources,^12^ as well as in engine particle emissions characterization^13^ and air quality monitoring.^14^ However, most of these studies have relied on visible or near-infrared wavelengths, due to its featureless profile, where absorption provides limited chemical information. The extension of PAS to the mid-infrared C–H stretching region, as demonstrated in this work, enables chemically specific aerosol characterization based on fundamental molecular vibrational transitions and opens new opportunities for quantitative hydrocarbon aerosol measurements.

Historically, PAS has relied on high-power mid-infrared sources—CO_2_ lasers, optical parametric oscillators, or quantum cascade lasers (QCLs)—to achieve adequate sensitivity.^15,16^ While effective for practical applications, these approaches face fundamental limitations: CO_2_ lasers are bulky, optically and mechanically complex, offer only discrete wavelengths, and are ill-suited for field deployment;^17,18^ QCLs require high drive currents and complex thermal management;^19,20^ and all demand external power normalization to correct for wavelength-dependent laser output.^21,22^ These complications have hindered the development of compact, field-deployable instruments capable of acquiring quantitative absorption spectra across the chemically informative C–H stretching region.

Interband cascade lasers (ICLs) have emerged as transformative sources for mid-infrared spectroscopy.^23,24^ Unlike QCLs, which rely on intersubband transitions, ICLs generate photons via interband electronic transitions, offering inherently broader gain, lower threshold currents, and continuous-wave operation at room temperature with milliwatt-level output.^25–27^ When configured in an external cavity (EC)-ICL, these devices provide narrow line width (sub nm) and wide tunability—typically 250–350 nm in the 3–4 μm range—enabling comprehensive investigation of aliphatic C–H stretching bands.^23^ Critically, their low power consumption and compact footprint make them ideal for integrated sensor systems.^28^ In parallel, traditional approaches employ separate power monitors, gas cells with known absorption, or first-principles calculation of cell constants.^29,30^ Each has limitations: power monitors require precise knowledge of the detector’s spectral responsivity, and any measurement uncertainty in the monitor propagates into the calculated absorption coefficients; gas cells demand frequent validation; and calculable cell constants rely on poorly constrained relaxation parameters for complex aerosols.^30^ Simultaneous measurement of a reference gas and analyte aerosol in identical optical conditions would elegantly circumvent these issues, yet such dual-cell configurations have not been demonstrated for mid-infrared aerosol characterization.

Here, we introduce a dual-cell photoacoustic system coupled with an EC-ICL tunable from 3.30 to 3.55 μm. We demonstrate a wavelength-dependent transfer function (TF) derived from a reference gas (propane) of known absorption, which normalizes the analyte aerosol signal to yield quantitative absorption coefficients in absolute units (cm^–1^). This approach inherently compensates for all instrumental variations—laser power, beam alignment, microphone sensitivity, and cell constant—without any external calibration protocol.

We validate the method using diethyl-hexyl-sebacate (DEHS) aerosol as a model aliphatic hydrocarbon system. The DEHS particles, with their well-characterized C–H stretching spectrum, enable direct comparison with bulk-phase ellipsometry measurements of the complex refractive index—a validation framework rarely applied in aerosol photoacoustic studies.^29^ Our results demonstrate agreement within 2.1% between photoacoustic-derived absorption and Rayleigh-theory predictions from ellipsometry, establishing the accuracy of the transfer function methodology.

To the best of our knowledge, this work represents the first demonstration of quantitative aerosol absorption spectroscopy in the mid-infrared C–H stretching region using a dual-cell EC-ICL platform. The approach combines three advances: (1) the first application of a dual-cell configuration for simultaneous reference gas and analyte aerosol measurement; (2) quantitative validation against bulk ellipsometry across a continuous mid-IR spectral window; and (3) a simplified, calibration-free methodology suitable for field deployment. The ability to perform quantitative, calibration-free mid-infrared absorption spectroscopy of hydrocarbon aerosols has broad relevance across several research and application areas. In atmospheric science, it enables real-time characterization of combustion-derived aerosols for source apportionment studies and provides wavelength-resolved absorption data essential for improving radiative forcing estimates. In industrial settings, the technique offers potential for hydrocarbon leak detection and emission monitoring with minimal maintenance requirements. The approach is also relevant for health-related investigations, where chemical-specific aerosol characterization can support exposure assessment and epidemiological studies. The dual-cell EC-ICL system described here is designed to address such applications by combining calibration-free operation, compact form factor, and real-time data acquisition. These capabilities position the technique for transformative applications in environmental monitoring, industrial safety, and climate-relevant aerosol characterization.

## Methodology

### EC-ICL Laser

A schematic of the experimental setup is shown in Figure 1. The EC-ICL (Sacher Lasertechnik, Germany) employs a Littman–Metcalf cavity^31^ in which a diffraction grating and prism provide wavelength selection. Tuning from 3.30 to 3.55 μm is achieved by simultaneously rotating and translating the grating via a stepper motor (minimum step 0.05 nm), maintaining subnanometre resolution across the range. The zeroth-order output beam (collimated, 3 mm diameter) is used for all measurements. The laser cavity integrates a gain chip (maximum output ∼ 4 mW at 3.4 μm) and operates in continuous-wave (cw) mode with the heat sink temperature stabilized at 18 °C ± 0.01 °C by a Peltier controller.

**Figure 1:**
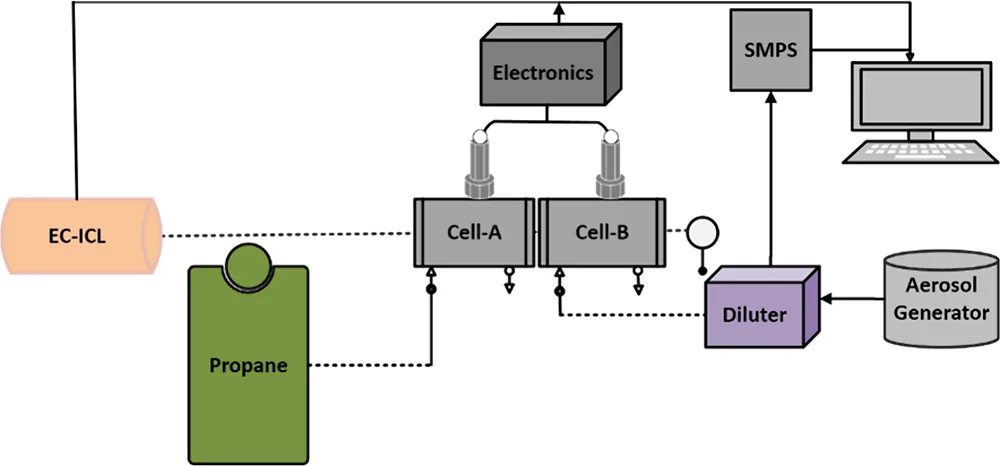
Schematic diagram of the dual-cell photoacoustic system. The EC-ICL beam passes sequentially through Cell A (reference) and Cell B (aerosol), with AR-coated windows minimizing losses. Laser parameters and microphone signals from both cells are processed by customized electronics.

Wavelength calibration of the laser is performed using a gas cell filled with low-pressure propane (10 mbar) and a reference wavemeter (Bristol 621B, accuracy ± 0.2 pm). The laser line width is specified as <0.005 nm (instrument-limited). Output power is monitored by a thermal power sensor (Thorlabs S401C) placed after the dual-cell assembly; power varies with wavelength from 1.8 to 4.0 mW (Figure 2).

**Figure 2:**
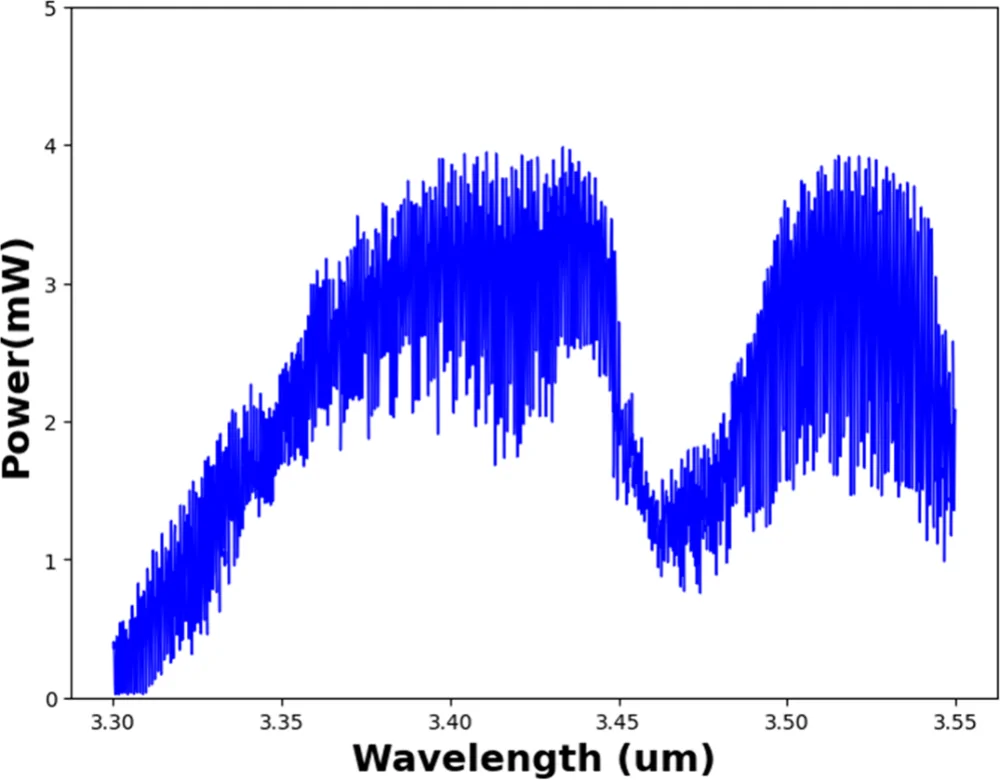
CW power spectrum of the EC-ICL as a function of wavelength, measured at a laser diode current of 225 mA, and temperature of 18 °C.

Laser intensity modulation was achieved by direct current modulation applied to the laser driver (Sacher MLD-1000) via a DC-coupled BNC input. A square wave at a frequency of 4.410 kHz with 50% duty cycle is generated by a custom electronics module that integrates microphone amplifiers, lock-in signal detection, and cell temperature controllers and simultaneously provides the reference signal for lock-in detection. The modulation depth is set to 80% of the maximum current to maintain single-mode operation while maximizing the acoustic signal.

### EC-ICL-Based Dual PA Cell Configuration

The dual-cell assembly comprises two identical differential photoacoustic cells (Cell A and Cell B) arranged in series along the optical axis (Figure 2). Each cell is machined from stainless steel and contains a cylindrical acoustic resonator (length 36 mm, diameter 4 mm) with buffer volumes at both ends to suppress flow noise.^32^ The resonator geometry was optimized using finite-element modeling (COMSOL Multiphysics) to yield a longitudinal resonance frequency of approximately 4.41 kHz with a quality factor *Q* = 15 under ambient conditions (air, 1 atm, 295 K). Both cells are equipped with identical electret microphones (Knowles EK-3029) positioned at the pressure antinode (midpoint of the resonator). The sensitivity of the developed dual-cell photoacoustic system was evaluated in terms of the minimum detectable optical absorption coefficient (MDOAC), which is the standard performance metric for photoacoustic aerosol instruments.^33^ The MDOAC was determined from the baseline noise of the photoacoustic signal using the conventional 3σ criterion, where the standard deviation of the detector signal recorded in the absence of absorbing aerosols was converted into an equivalent optical absorption coefficient using the experimentally determined wavelength-dependent transfer function. For a measurement integration time of 3 s, the system achieved an MDOAC of 0.8 Mm^1–^ at 3.34 μm, demonstrating high sensitivity for quantitative laboratory aerosol absorption measurements. The resonant photoacoustic cells employed in this work have also been previously characterized, demonstrating subppm gas detection sensitivity, with minimum detectable concentrations of 0.12 ppm for N_2_O and 0.42 ppm for NH_3_ under resonant operating conditions.^34^ The cell constant was determined by filling both cells with a certified mixture of 100 ppm propane in N_2_ and measuring the photoacoustic signal under known absorption conditions. The product MC (microphone sensitivity × cell constant) was found to be nearly constant over the tuning range. Optical access is provided by antireflection-coated ZnSe windows (Thorlabs WW81050-E3, transmission >95% at 3.3–3.5 μm) sealed with Viton O-rings. The windows are tilted by 2° relative to the optical axis to suppress etalon fringing. Gas inlets and outlets are positioned in the buffer volumes to allow continuous flow (0.5 L/min) while maintaining ambient pressure. The aerosol flow enters the cell perpendicular to the optical beam and passes through the resonator before exiting perpendicularly via a pump. This flow path minimizes particle impaction on the optical windows, while the transfer-function approach reduces the impact of common-mode optical losses that may arise from gradual window contamination.

The PA signal measured in Cell A when filled with a reference gas of known absorption is given by

1 $$S_{r}^{A}\left(\lambda \right)=P\left(\lambda \right)\times M\times C\times \alpha \left(\lambda \right)$$

where *P*(λ) is the modulated laser power, *M* is the microphone sensitivity (V/Pa), *C* is the cell constant (Pa·cm/W), and α­(λ) is the absorption coefficient of the reference gas (cm^–1^). We note that *P*(λ) represents the effective modulated optical power at the modulation frequency. Figure 2 shows the CW output power spectrum of the EC-ICL for reference. In the transfer-function methodology, the photoacoustic signal is measured under the actual modulation conditions; therefore, any wavelength dependence associated with the modulated optical power is inherently incorporated into the transfer function. The unknown wavelength-dependent power factor was eliminated by forming a transfer function from the reference measurement:

2 $$\mathrm{TF}\left(\lambda \right)=\frac{S_{r}^{A}\left(\lambda \right)}{\alpha \left(\lambda \right)}$$

This TF (V/cm^–1^) encapsulates all instrumental contributions to the photoacoustic signal in Cell A. Because the same laser and optical path are used for both cells, and because the cell properties are matched, the aerosol absorption coefficient can then be obtained from the Cell B signal as

3 $$\beta_{\mathrm{PA}}\left(\lambda \right)=\frac{S_{r}^{B}\left(\lambda \right)}{\mathrm{TF}\left(\lambda \right)}$$

Equation 3 yields β­(λ) in absolute units (cm^–1^) without requiring external power normalization, separate microphone calibration, or knowledge of the cell constant. The TF is determined once per measurement session or can be updated continuously by flowing reference gas through Cell A while measuring aerosols in Cell B. The latter can exclude the time dependence of cell parameters during the measurement. The validity of the transfer-function approach relies on the assumption that relaxation-induced differences in photoacoustic conversion efficiency between the propane reference gas and the DEHS aerosol are negligible. To evaluate this assumption, the lock-in phase responses of the two signals were compared at the operating modulation frequency of 4.410 kHz. No measurable wavelength-dependent phase lag was observed, indicating that relaxation-induced losses are negligible under the present experimental conditions. This conclusion is further supported by the excellent agreement between the photoacoustically derived absorption coefficients and the independent ellipsometry-based calculations.

Propane (C_3_H_8_, 99.5% purity, Linde) was chosen as the reference gas because its mid-infrared absorption spectrum in the 3.30–3.55 μm region is well characterized and dominated by the same C–H stretching vibrations present in hydrocarbon aerosols. Certified mixtures of 100 ppm propane in nitrogen were prepared using mass flow controllers (Bronkhorst) and verified by a reference photoacoustic cell prior to experiments. The absorption coefficient α­(λ) for propane at this concentration was taken from the Pacific Northwest National Laboratory (PNNL) infrared database,^35^ which provides absolute absorption cross sections (cm^2^/molecule) with an estimated uncertainty of ±2%.

Diethylhexyl sebacate (DEHS, C_26_H_50_O_4_, >97% purity, Sigma-Aldrich) was selected as the model hydrocarbon aerosol due to its well-defined aliphatic structure, low volatility, and widespread use as a calibration standard in aerosol PAS.^30^ DEHS aerosols were generated by using a collision-type atomizer (TOPAS ATM 220) operated with nitrogen carrier gas at 1 bar. The polydisperse output was diluted 10-fold with particle-free ambient air using a dilution system (PALAS VKL-10) to achieve stable number concentrations.

Prior to the measurements, the generated particle size distribution was determined and characterized using a scanning mobility particle sizer (SMPS, TSI 3938) consisting of an electrostatic classifier (TSI 3082) and a condensation particle counter (TSI 3750). The mobility diameter distribution was log-normal, with a geometric mean diameter of *d*_*g*_ = 199 nm, a total number concentration of *N* = 7.5 × 10^6^ cm^–3^, and a geometric standard deviation of σ_g_ = 1.831. The aerosol flow was introduced into Cell B at 0.5 L min^–1^. All measurements were repeated at least three times under identical conditions to assess measurement reproducibility. The relative standard deviation of the PA signals across repeated runs was below 2%, demonstrating good reproducibility of both the reference gas and aerosol measurements.

### Bulk Ellipsometry for Validation

To validate the quantitative accuracy of the photoacoustic-derived absorption coefficients, we independently measured the complex refractive index *m̃* = *n* + *ik* of bulk DEHS using spectroscopic ellipsometry. The ellipsometric mid-IR measurements were performed with a Semilab SE-2000 equipped with an FTIR-based infrared extension and a Mercury Cadmium Telluride detector, covering a wavenumber range of 600–7500 cm^–1^ (0.074–0.930 eV). Vibration isolation was provided by a pneumatic isolator, enabling stable measurements at the liquid surface.^36^ The acquisition parameters were set to 128 scans, a spectral resolution of 8 cm^–1^, and a mirror velocity of 0.5 cm/s. Measurements were performed at 3 angles of incidence: 65, 70, and 75° in a Petri dish, utilizing the horizontal sample position of the Semilab ellipsometer. The measured spectra were analyzed with the Semilab SEA software, with spectral fitting performed over the energy range of 0.115–0.6 eV. From the bulk refractive index, the absorption coefficient of a collection of spherical particles in the Rayleigh regime (diameter ≪ λ) can be computed as

4 βRayleigh(λ)=6πλ·Im(m̃2−1m̃2+2)·Cvol

where *m̃* = *n* + *ik* is the complex refractive index relative to the medium and *C*_vol_ is the volume concentration of aerosol (cm^3^ aerosol per cm^3^ air). The volume concentration was obtained from SMPS measurements assuming spherical particles.

Comparison between β_PA_ (λ) (from eq 3) and β_Rayleigh_ (λ) (from eq 4) provides a direct validation of the photoacoustic methodology. For polydisperse DEHS particles used here, the Rayleigh criterion π*d*/λ ≪ 1 is satisfied across the measurement range, justifying the approximation. For the calculation of the OAC coefficient of polydisperse aerosols using (*n*, *k*) values for comparison with photoacoustic-derived OAC results, we calculated the absorption efficiency at each size bin of the SMPS independently for the complete wavelength tuning range and summed them.

## Results and Discussions

The resonance characteristics of both cells are listed in Figure 3. Cell A exhibits a peak resonance at 4.410 kHz with a quality factor *Q* = 14.11 and 14.34 for Cells A and B, respectively. The close alignment of acoustic resonance frequencies enables simultaneous operation at a single modulation frequency (4.410 kHz) with less than 2.45% in signal amplitude for either cell, simplifying the measurement protocol without compromising sensitivity.

**Figure 3:**
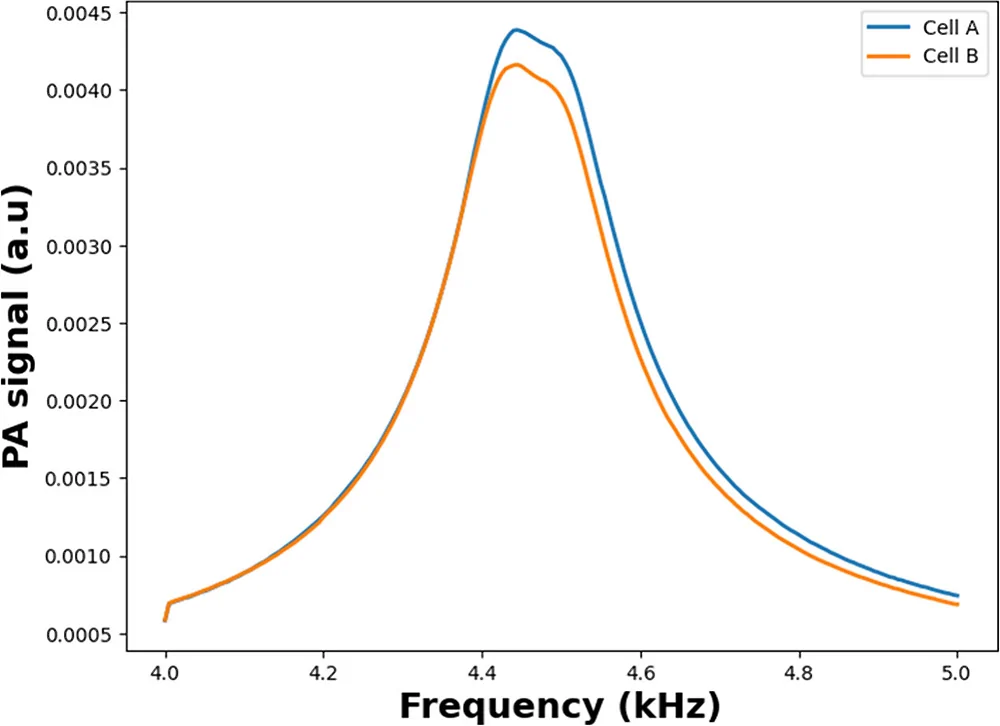
Resonance frequency measurements for PA Cells A and B. The near-identical resonance frequencies allow single-frequency operation.

Figure 4 compares the photoacoustic spectra of propane (100 ppm in N_2_) acquired simultaneously from Cell A and Cell B. Both spectra exhibit the characteristic C–H stretching features of propane: the prominent doublet at 3.37–3.40 μm (asymmetric CH_3_ stretches) and the weaker features near 3.47 μm (symmetric CH_3_ stretches).^37^ The spectral positions and relative intensities are in excellent agreement between cells, confirming that the dual-cell configuration preserves spectral fidelity. The amplitude difference (∼15% lower in Cell B) primarily arises from absorption in Cell A and optical losses at the AR-coated window between cells and was accounted for by normalizing the Cell B signal with the transfer function and the measured window transmission.

**Figure 4:**
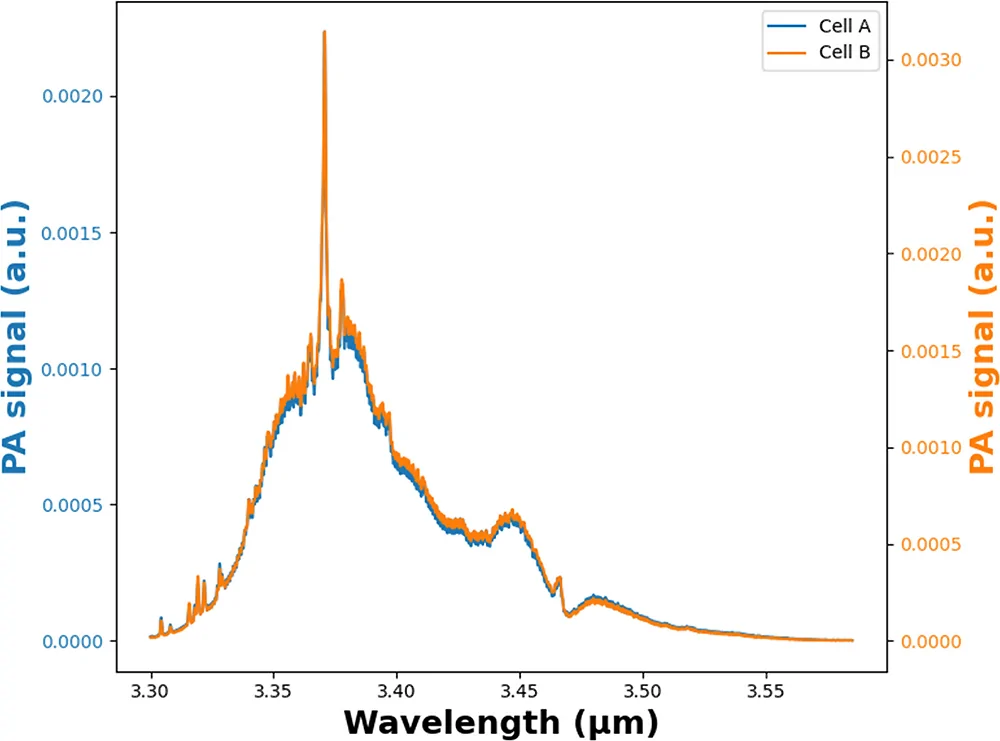
Photoacoustic spectra of propane (100 ppm in N_2_) measured simultaneously in Cell A and Cell B. The excellent agreement in peak positions confirms spectral fidelity across the dual-cell configuration.

Validation against the PNNL reference database is presented in Figure 5. The PA spectrum from Cell A (left axis) is overlaid with the PNNL absorption cross-section (right axis) scaled to the same concentration and path length. The measured spectrum reproduces all major features of the reference with a mean absolute deviation of <2% across the 3.30–3.55 μm range

**Figure 5:**
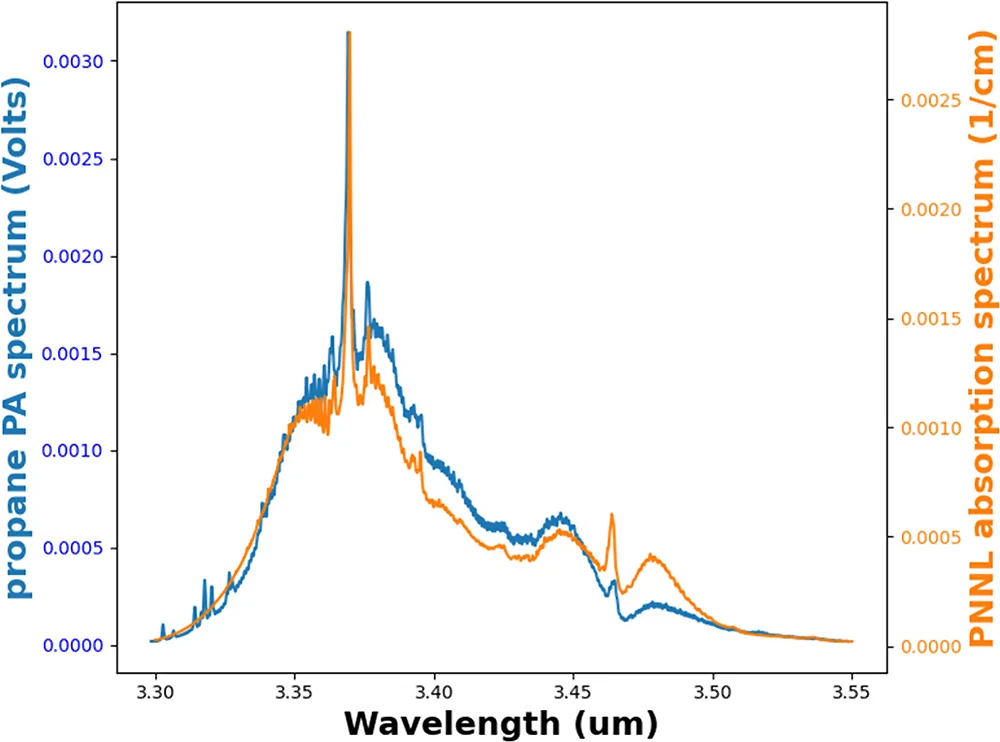
Comparison of measured propane PA spectrum from Cell A (left) with PNNL reference data (right axis, 100 ppm). The close agreement validates the accuracy of the reference channel.

The transfer function derived from Equation 2 is shown in Figure 6. The TF exhibits a smooth wavelength dependence dominated by the laser power profile (Figure 2) and the window transmission with no sharp features. The smoothness of the TF confirms that no additional wavelength-dependent instrumental artifacts distort the measurement.

**Figure 6:**
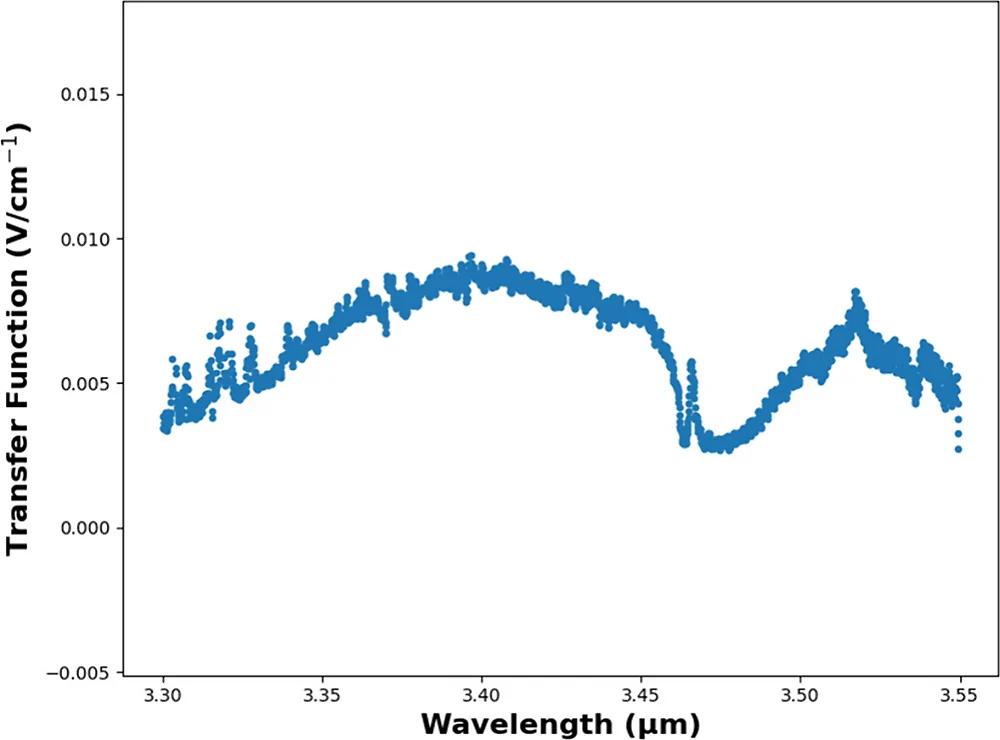
Transfer function TF derived from Cell A propane measurements using eq 2.

### Size distribution and quantitative DEHS aerosol spectra

Figure 7 presents the raw photoacoustic spectrum of DEHS aerosols measured in Cell B. The spectrum reveals distinct absorption features characteristic of aliphatic C–H stretching vibrations. The dominant absorption complex centered at 3.38–3.42 μm (2950–2920 cm^–1^) arises from C–H asymmetric stretching modes of CH_2_ and CH_3_ groups, while the secondary feature at 3.48–3.52 μm (2870–2840 cm^–1^) corresponds to C–H symmetric stretching modes.^37^ These assignments are consistent with the established infrared spectroscopy of long-chain aliphatic compounds.^38^ The absence of detectable absorption at ∼3.30 μm (3030 cm^–1^)—a region associated with aromatic C–H stretches—confirms that DEHS behaves as a purely aliphatic hydrocarbon aerosol, consistent with its molecular structure as a long-chain diester (C_26_H_50_O_4_). This spectral purity validates DEHS as an appropriate model system for validating the quantitative methodology.

**Figure 7:**
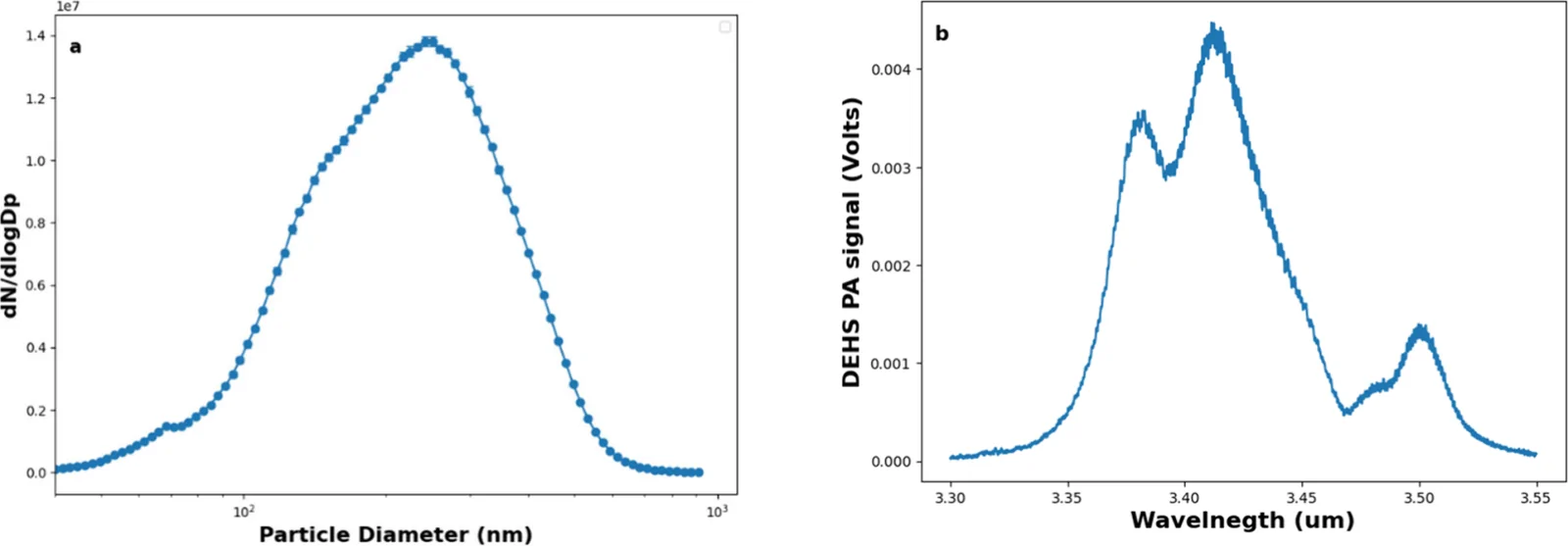
(a) Size distribution of DEHS aerosols using SMPS; (b) Raw photoacoustic spectrum of DEHS aerosols measured in Cell B (wavelength resolution 0.05 nm, averaging time 800 ms).

### 3.2 Validation against Bulk Ellipsometry

Applying the wavelength-dependent transfer function (eq 2) to the raw DEHS spectrum yields the optical absorption coefficient (OAC) in absolute units (cm^–1^). The spectrum retains the characteristic aliphatic features observed in Figure 8 but is now quantitatively calibrated. Validation of the quantitative PA results was performed by comparison with absorption coefficients calculated from bulk ellipsometry measurements of DEHS complex refractive index, employing the methodology described in in previous section. Figure 8 presents absolute OAC computed from ellipsometry data using eq 4, accounting for the full polydisperse size distribution.

**Figure 8:**
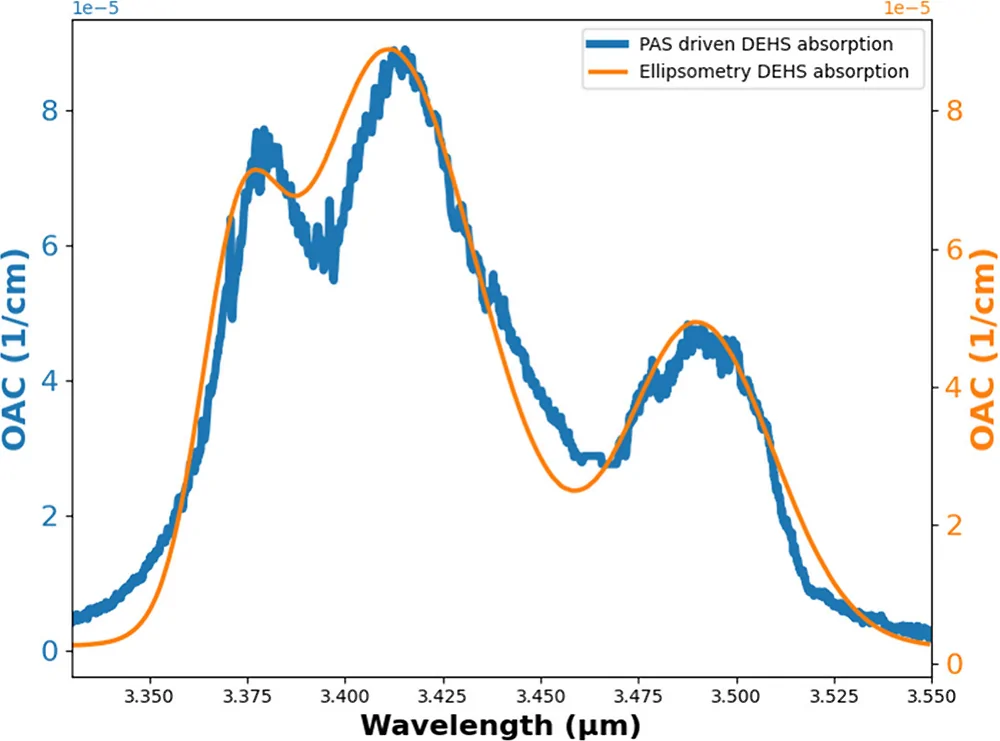
Comparison of photoacoustic- driven absorption coefficient with Rayleigh-theory prediction from bulk ellipsometry.

The agreement between the two independent determinations is excellent across the entire spectral range. The mean relative deviation, calculated as

$$\mathrm{mean relative deviation}=\frac{1}{N}\overset{N}{\underset{i=1}{\sum }}\frac{\left|\beta_{\mathrm{PA}}\left(\lambda_{i}\right)-\beta_{\mathrm{Rayleigh}}\left(\lambda_{i}\right)\right|}{\beta_{\mathrm{Rayleigh}}\left(\lambda_{i}\right)}=0.021$$

yields 2.1% average difference, well within the combined experimental uncertainties (PA: ±2%, ellipsometry: ±5%, size distribution: ±3%). The residual wavelength-dependent deviations show no systematic trend, confirming that the transfer function approach successfully eliminates instrumental artifacts. The dual-cell transfer-function approach demonstrated here establishes a practical methodology for quantitative mid-infrared aerosol absorption spectroscopy while utilizing a highly sensitive resonant photoacoustic platform. The developed system achieved a minimum detectable optical absorption coefficient (MDOAC) of 0.8 Mm^-1^ at 3.34 μm for a 3 s integration time, confirming its capability for quantitative laboratory aerosol absorption measurements. Consequently, the proposed methodology is well suited for chemically specific characterization of hydrocarbon aerosols, combustion emission studies, aerosol source apportionment, and validation of aerosol optical models. The methodology also provides a foundation for future atmospheric applications; however, the practical detection limit under ambient conditions additionally depends on aerosol optical properties, particle concentration, and sampling conditions. This work represents the first demonstration of such a method specifically designed for aerosol measurements, with validation against bulk ellipsometry yielding 2.1% agreement—a quantitative benchmark rarely achieved in aerosol photoacoustic studies. The EC-ICL platform enables compact, field-deployable instrumentation without compromising spectral resolution, with direct implications for source apportionment of combustion aerosols, radiative forcing assessment, industrial leak detection, and health effects studies. Integration with scattering measurements via an integrating sphere module would further enable complete optical characterization, including retrieval of single-scattering albedo for climate-relevant applications.

## Conclusion

We have demonstrated, for the first time, a dual-cell photoacoustic spectroscopy approach for quantitative characterization of hydrocarbon aerosols in the mid-infrared C–H stretching region (3.30–3.55 μm). The key innovation—a wavelength-dependent transfer function derived from simultaneous reference gas measurements—enables determination of absolute aerosol absorption coefficients without external power normalization or frequent calibration. Validation against bulk ellipsometry confirms the accuracy of the method, with 2.1% mean agreement across the entire spectral range—the most comprehensive validation reported for mid-infrared aerosol PAS to date.

The EC-ICL laser source, with its narrow line width, wide tunability, and low power consumption, proves ideally suited for this application, enabling compact instrument design without sacrificing spectral resolution or sensitivity. The dual-cell configuration, with identical resonators and a matched microphone response, ensures that the transfer function accurately captures all wavelength-dependent instrumental factors.

The system described here is suitable for laboratory investigations of hydrocarbon aerosol optical properties, atmospheric studies of combustion-derived aerosols, source apportionment research combining absorption spectroscopy with chemical analysis, and climate-related studies requiring wavelength-resolved aerosol absorption coefficients for radiative transfer modeling. The methodology may also support industrial emission monitoring and hydrocarbon leak detection through quantitative chemically specific aerosol measurements. This work establishes a new paradigm for quantitative aerosol absorption spectroscopy in the mid-infrared fingerprint region with direct applications in atmospheric monitoring, climate science, and industrial safety. Future developments will focus on expanding spectral coverage, integrating scattering measurements for complete optical characterization, and validating the approach for diverse aerosol types relevant to environmental and health effect studies.
